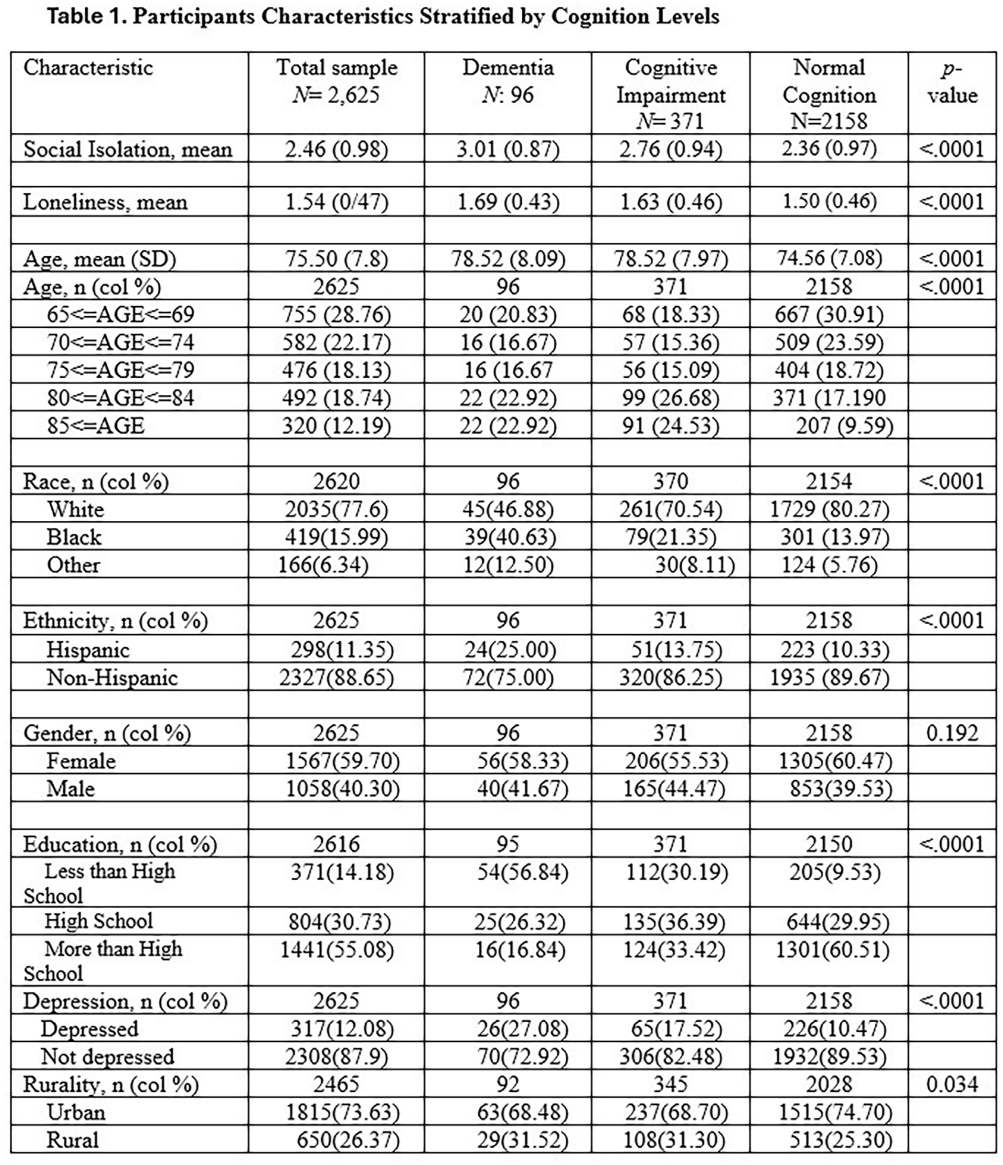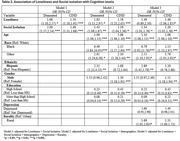# Social Isolation and Loneliness in Varied Levels of Cognition Among Racially/Ethnically Diverse Older Americans

**DOI:** 10.1002/alz.095749

**Published:** 2025-01-09

**Authors:** Ayse Malatyali, Tom Cidav, Rui Xie, Ladda Thiamwong, Lisa Ann Kirk Wiese

**Affiliations:** ^1^ University of Central Florida, 12201 Research Parkway, Suite 300 Orlando, FL USA; ^2^ U.S. Department of Veterans Affairs, Philadelphia, PA USA; ^3^ University of Central Florida, Orlando, FL USA; ^4^ University of Central Florida, Orlando, FL, 32826, FL USA; ^5^ C.E. Lynn College of Nursing, Florida Atlantic University, Boca Raton, FL USA

## Abstract

**Background:**

Social isolation and loneliness affect 20%‐30% of older Americans, increasing their risk of depression, admission to nursing homes, early disability, and mortality. This study examines the association of social isolation and loneliness with cognitive impairment‐not‐dementia (CIND) and dementia among racially/ethnically diverse older Americans.

**Method:**

We analyzed data from the Health Retirement Study (2020 interviews) using logistic regression analyses with cognition as a response. Our sample consisted of 2,625 participants (age > 64). Measures included the 27‐item HRS cognition scale, five‐item social isolation scale, UCLA loneliness scale, and Center for Epidemiological Studies Depression scale.

**Result:**

The characteristics of our sample were: mean age of 75.5 years, Blacks (16%), Hispanics (11.4%), women (59%), College educated (55%), and residing in rural areas (26.3%). Participants who experienced loneliness were 40% more likely to have cognitive impairment and 20% more likely to have dementia. The likelihood of having cognitive impairment was 20% higher among socially isolated participants than those without social isolation. However, there was no significant association between social isolation and dementia. The relationship of depressive symptoms with CIND or dementia was also non‐significant. Black participants were about seven times more likely to have CIND and two times more likely to have dementia than Whites. Hispanic participants had nearly three times higher risk of CIND than non‐Hispanics, but their risk of dementia was non‐significant. Further interaction analyses showed no statistically significant difference in social isolation or loneliness with CIND or dementia risk by race or ethnicity. The odds of having CIND were significantly higher in men (20%) than women and in rural participants (31%) than urban participants. Conversely, the association of gender groups or rurality with dementia was non‐significant. The risk of CIND and dementia was significantly less in participants with a high school degree and above.

**Conclusion:**

These findings support research on the importance of social connectedness in cognitive aging. Further investigation into temporal trends in social isolation and loneliness regarding the changes in cognitive trajectories is needed.